# A Case of Pheochromocytoma With Coagulation Necrosis Due to Hypertensive Crisis Aggravated by Contrast-Enhanced CT Scan and Negative 123I-Metaiodobenzylguanidine (MIBG) Scintigraphy

**DOI:** 10.7759/cureus.56878

**Published:** 2024-03-25

**Authors:** Ai Kobayashi, Yuki Ishinoda, Asuka Uto, Sho Ogata, Naoki Oshima

**Affiliations:** 1 Department of Endocrinology, National Defense Medical College, Saitama, JPN; 2 Department of Laboratory Medicine, National Defense Medical College, Saitama, JPN; 3 Department of Nephrology, National Defense Medical College, Saitama, JPN

**Keywords:** contrast-enhanced ct scan, 123i-metaiodobenzylguanidine scintigraphy, coagulative necrosis, hypertensive crisis, pheochromocytoma

## Abstract

^123^I-metaiodobenzylguanidine (^123^I-MIBG) scintigraphy is a highly sensitive and specific imaging test for the diagnosis of pheochromocytoma. Typical pheochromocytomas are positive on ^123^I-MIBG scintigraphy; however, cases of paragangliomas eliciting negative results have been reported. We encountered a case of hypertensive crisis resulting in extensive coagulative necrosis of a pheochromocytoma and negative findings on ^123^I-MIBG scintigraphy. A 50-year-old Japanese female presented with an acute onset of vomiting, epigastralgia, and abdominal pain. Immediately after contrast-enhanced CT, the patient developed respiratory failure and was intubated. The CT scan revealed a 5-cm left adrenal mass, and a pheochromocytoma crisis was suspected. The patient’s condition stabilized following phentolamine administration. Regarding the assessment for pheochromocytoma, plasma metanephrine levels were not markedly increased, and ^123^I-MIBG scintigraphy was negative. However, a histological examination of the left adrenal mass revealed extensive coagulative necrosis of the entire adrenal mass, comprising trabecular and alveolar growth of large polygonal cells that were immunopositive for chromogranin A/synaptophysin, thereby suggesting a diagnosis of pheochromocytoma. There have been three reported cases of ^123^I-MIBG scintigraphy-negative pheochromocytomas because of pure avascular necrosis without hemorrhage or rupture. To the best of our knowledge, this is the first reported case of massive tumor necrosis due to hypertensive crisis exacerbated after contrast-enhanced CT imaging. In conclusion, pheochromocytoma cannot be ruled out even with negative findings on ^123^I-MIBG scintigraphy. Accordingly, clinical judgment must be made based on a comprehensive assessment of the clinical course and pathological diagnosis, especially for cases involving a hypertensive crisis.

## Introduction

Pheochromocytomas arise from chromaffin cells of the neural crest, and their pathogenesis is characterized by intermittent surges in catecholamine levels, eliciting symptoms such as hypertension, headaches, palpitations, and sweating [1]. Pheochromocytoma is considered a differential disease in cases of familial history or history of pheochromocytoma, catecholamine excess, refractory or paroxysmal hypertension, and adrenal incidentalomas [2]. Pheochromocytoma is diagnosed based on the elevated serum and urine levels of catecholamine or its metabolites, as well as the detection of tumors on imaging tests. Chromophilic cells not only produce catecholamines but also possess noradrenaline transporters (NATs) that facilitate the intracellular uptake of catecholamines (especially noradrenaline). Metaiodobenzylguanidine (MIBG) is a guanidine analog of noradrenaline that enters chromaffin cells via NATs. Subsequently, MIBG is stored in neurosecretory granules via vesicular monoamine transporters [3,4]. Based on these principles, ^123^I-metaiodobenzylguanidine (^123^I-MIBG) scintigraphy is widely employed to diagnose pheochromocytomas, exhibiting excellent sensitivity and specificity (88 and 84-100%, respectively) [5]. However, rare cases of pheochromocytoma negative on ^123^I-MIBG scintigraphy have been documented, potentially attributed to reasons such as extremely small tumors, predominantly cystic components, necrosis and hemorrhage, drugs that inhibit MIBG transfer to chromaffin cells, and succinate dehydrogenase B (SDH) gene mutations [6-8]. Herein, we describe a case of pheochromocytoma wherein a hypertensive crisis led to coagulation necrosis of the tumor, with normal catecholamine levels and negative results on ^123^I-MIBG scintigraphy in the preoperative examination.

## Case presentation

**Figure 1:**
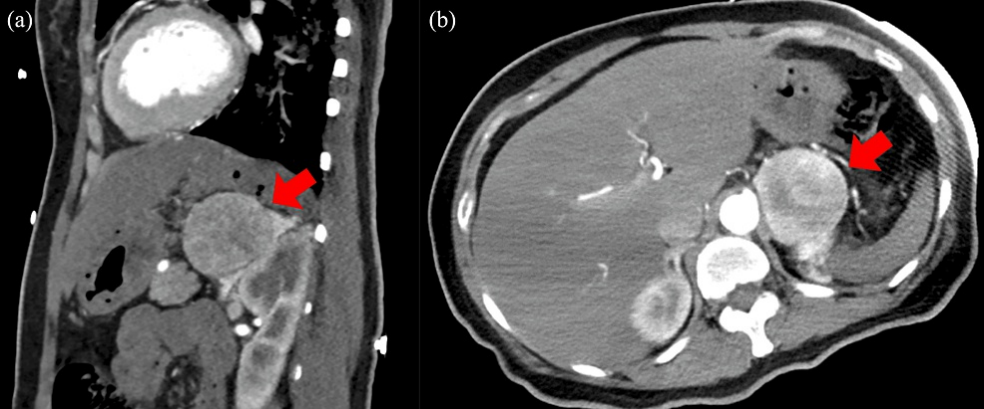
Contrast-enhanced CT scan performed at a previous hospital Physical findings of the patient. A 52-mm mass contiguous with the left adrenal gland is seen. (a) Sagittal section and (b) horizontal section.

A 50-year-old Japanese female presented with intermittent episodes of headaches, nausea, vomiting, fatigue, and palpitations for three years. Additionally, she experienced epigastralgia and right abdominal pain, accompanied by severe vomiting. Hence, she visited a hospital. Upon admission, her blood pressure was 204/146 mmHg; consequently, continuous intravenous diltiazem was administered. A contrast-enhanced CT revealed a 52-mm mass in the left adrenal gland, which showed contrast in the artery-dominant phase (Figure 1).

Pulmonary edema was also observed. However, the patient’s respiratory condition rapidly deteriorated after undergoing a contrast-enhanced CT. Accordingly, she was placed on a ventilator and transferred to our hospital for multidisciplinary and intensive care management. The patient had been diagnosed with type 2 diabetes three years prior and had been taking three oral hypoglycemic agents, with hemoglobin A1C (HbA1c) ranging within 10%. The patient had no history of hypertension, was an occasional drinker, and had no history of smoking. Regarding the family history, the patient had a paternal history of lung cancer and a maternal history of diabetes and hypertension. Examination upon admission revealed the following: Glasgow Coma Scale score, E2VTM5; height, 152 cm; weight, 51 kg; body mass index, 22.0 kg/m^2^; temperature, 38.8°C; pulse, 126/min; blood pressure, 149/110 mmHg at 15 mg/h of diltiazem; and oxygen saturation, 100% (inspiratory oxygen fraction, 0.8). The blood test findings were as follows: aspartate transaminase/alanine aminotransferase, 190/75 U/L; gamma-glutamyl transpeptidase, 137 U/L; and creatine 1.42 mg/dL (indicating renal dysfunction). The leukocyte, erythrocyte, and platelet counts were 28,700/μL, 5.4 million/μL, and 638,000/μL, respectively. The blood glucose level was 833 mg/dL (Table 1), and her urine was negative for ketones.

**Table 1:** Laboratory findings on hospitalization PT, prothrombin time; APTT, activated partial thromboplastin time; FDP, fibrinogen/fibrin degradation products; T-bil, total-bilirubin; AST, aspartate aminotransferase; ALT, alanine aminotransferase; ALP, alkaline phosphatase; γ-GTP, γ-glutamyl transpeptidase; LDH, lactate dehydrogenase; TP, total protein; Alb, albumin; BUN, blood urea nitrogen; Cr, creatinine; HbA1c, hemoglobin A1c; Na, Sodium; K, potassium; Cl, chloride; CK, creatinine kinase; CRP, C-reactive protein; TSH, thyroid stimulating hormone; FT4, free thyroxine; BNP, brain natriuretic peptide.

Laboratory examinations	Values	Unit	Reference range
Red blood cells	540	10^3^/μL	435–555
Hemoglobin	16.5	g/dL	13.7–16.8
White blood cells	28.7	10^3^/µL	3.3–8.6
Neutrophil	95.0	%	40.0–70.0
Lymphocyte	3.9	%	20.0–50.0
Monocyte	0.9	%	2.0–9.0
Eosinophil	0.0	%	1.0–6.0
Basophil	0.2	%	0.0–2.0
Platelets	63.8	10^3^/μL	15.0–40.0
PT	10.1	sec	9.9–11.8
APTT	24.4	sec	24.0–32.0
D dimer	7.9	µg/mL	0.0–1.0
FDP	10.0	µg/mL	0.0–5.0
T-bil	0.3	mg/dL	0.2–1.2
AST	190	IU/L	8–30
ALT	75	IU/L	5–35
ALP	145	IU/L	38–113
γ-GTP	137	IU/L	7–70
LDH	483	IU/L	100–225
TP	7.1	g/dL	6.5–8.2
Alb	4.0	g/dL	3.8–5.2
BUN	23	mg/dL	8–20
Cr	1.42	mg/dL	0.61–1.13
Glucose	833	mg/dL	65–110
HbA1c	11.0	%	4.6–6.2
Na	130	mEq/L	135–147
K	5.4	mEq/L	3.5–5.0
Cl	91	mEq/L	98–108
CK	104	IU/L	0.0–160
CRP	< 0.3	mg/dL	0.0–0.3
TSH	1.40	µIU/mL	0.61–4.68
FT4	0.94	ng/dL	0.76–1.65
Troponin-I	577.5	pg/mL	0.0–28.0
BNP	69.5	pg/mL	0.0–18.4

After admission, her systolic blood pressure rapidly dropped to 50 mmHg, and continuous intravenous noradrenaline therapy was initiated. An echocardiography revealed 20% cardiac contractility with pulmonary edema, suggestive of acute heart failure. However, a comprehensive cardiology examination did not indicate myocardial infarction or pericarditis; therefore, catecholamine-induced cardiomyopathy was suspected. Additionally, given the severe blood pressure fluctuations and the presence of a left adrenal mass, a pheochromocytoma crisis was strongly suspected. Therefore, intravenous phentolamine was administered, which ameliorated the abnormal blood pressure fluctuations. Cardiac contractility recovered to >60% within a few hours of phentolamine administration, and blood pressure stabilized the day after admission without administration of inotropic or pressor drugs. The patient was extubated on the third day of admission, transferred from the ICU to the general ward on day six, and underwent a detailed examination for pheochromocytoma from day seven.

There was no apparent catecholamine excess detected in the blood or 24-hour urine fractionated metanephrine on days seven and eight, as the data were less than three times the upper limit of normal values (Table 2).

**Table 2:** Clinical laboratory results of catecholamines

Laboratory examinations	Values	Unit	Reference range
24-h urinary catecholamine (day seven)			
Adrenaline	60.8	μg/day	3.4–26.9
Noradrenaline	556.0	μg/day	48.6–168.4
Dopamine	1,712.2	μg/day	365.0–961.5
Metanephrine	0.30	mg/day	0.05–0.20
Normetanephrine	0.94	mg/day	0.10–0.28
Plasma metanephrine (day eight)			
Metanephrine	47	pg/mL	130 or less
Normetanephrine	299	pg/mL	506 or less
24-h urinary catecholamine (day 19)			
Adrenaline	14.4	μg/day	3.4–26.9
Noradrenaline	148.1	μg/day	48.6–168.4
Dopamine	1,079.8	μg/day	365.0–961.5
Metanephrine	0.16	mg/day	0.05–0.20
Normetanephrine	0.29	mg/day	0.10–0.28

**Figure 2:**
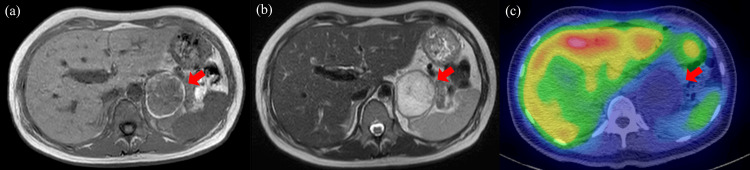
MRI and 123I-MIBG scintigraphy findings of the left adrenal mass (a) Simple sagittal T1-weighted MRI indicates a high signal area at tumor margins and a low signal area within the mass. (b) Contrast-enhanced MRI T2-weighted images show a low signal at the limbus and a high signal in the interior. (c) 123I-MIBG scintigraphy shows no accumulation at the mass.

An MRI on day five revealed coagulation necrosis of a substantial portion of the tumor (Figures 2a, 2b). Additionally, ^123^I-MIBG scintigraphy revealed no uptake by the tumor mass (Figure 2c).

**Figure 3:**
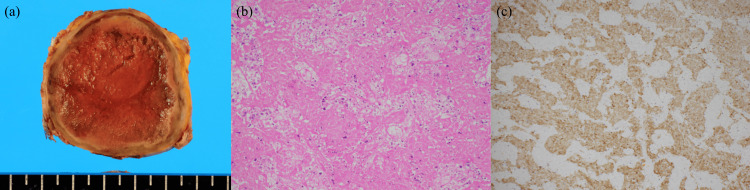
Pathological examination of the resected adrenal mass (a) The mass is 53×47×45 mm, and its cut surface exhibits a brown-colored, friable, solid mass with thickened fibrous capsule. Lines on a scale in Figure 3 indicate 5 mm each. (b) The mass shows massive coagulative necrosis, with trabecular and nested growth of polygonal tumor cells obscurely suggested. The tumor cells do not display marked nuclear atypia or obvious mitotic figures (HE, 40× in an objective lens). (c) The tumor cells appear diffusely immunopositive for chromogranin A (diaminobenzidine, 40× in an objective lens).

Accordingly, the imaging findings were attributed to coagulation necrosis of the tumor because of the pheochromocytoma crisis. On day 26, a laparoscopic left adrenalectomy was performed. The resected adrenal mass was 53×47×45 mm and grossly appeared as a well-circumscribed, brownish-toned, solid mass (Figure 3a). No significant intraoperative blood pressure changes were observed. Histological examination revealed extensive coagulative necrosis in almost the entire mass, with a small number of normal adrenal cortical cells in the periphery. The necrotic mass comprised large polygonal cells with round nuclei, displaying trabecular or nested proliferation (Figure 3b). Based on immunohistochemistry findings, neoplastic cells were diffusely positive for both chromogranin A and synaptophysin and negative for S100 protein (Figure 3c) and pankeratin.

These features were consistent with necrotic pheochromocytoma; however, further staging could not be performed because the majority of the tumor was necrotic. The postoperative course was uneventful, and the patient was discharged on day 37. Blood glucose levels showed good progress, necessitating only monotherapy. The patient is alive and will continue to be followed up with functional and imaging tests for the remainder of her life.

## Discussion

Pheochromocytoma crisis is a clinically important endocrine emergency, with a mortality rate as high as 35% [9]. Newell et al. defined pheochromocytoma crisis based on the following four signs: multiple organ failure, severe blood pressure abnormalities (hypertension or hypotension), high fever, and encephalopathy presenting as pheochromocytoma multisystem crisis (PMC) [10]. PMC is often difficult to diagnose in the acute phase because of its diverse clinical presentations. In the current case report, the patient presented with acute abdominal pain, vomiting, dyspnea, and hypertension, accompanied by acute exacerbation of respiratory failure immediately after contrast-enhanced CT. We suspected pheochromocytoma based on the presence of an adrenal mass and disease exacerbation following the use of contrast media. The blood pressure stabilized after intravenous injection of phentolamine mesylate, which strongly suggested a pheochromocytoma. The patient underwent laparoscopic adrenalectomy, and light microscopic findings revealed necrosis of the whole tumor, with peculiar “ghostlike” cellular and structural features, along with neuroendocrine immunophenotype morphology. There were no viable tumor cells, and the normal adrenal cortex was adjacent to the necrotic area, which suggested a pheochromocytoma arising within the adrenal gland. Although it has been reported that catecholamine elevation does not occur in patients with pheochromocytoma receiving currently available nonionic and hypoosmotic contrast media [11], the attached document states that contrast media should not be used in these patients except as necessary for diagnosis. A limitation of the current case is that a clear causal relationship between the contrast media and disease exacerbation remains unclear.

Pheochromocytomas, whether benign or malignant, are often relatively large, with CT pixel values frequently >20 Hounsfield units (HU) because of necrosis, cysts, hemorrhage, and calcification [12]. In addition to CT, MRI and nuclear medicine have been employed for imaging adrenal tumors. CT is generally considered the most feasible diagnostic modality for adrenal lesions in terms of spatial resolution, while MRI affords a high contrast resolution. In the current case, the adrenal tumor was ~5 cm in size at presentation and had a CT pixel value of ~30 HU. Although staining in the early contrast phase was consistent with a pheochromocytoma, it was atypical considering the homogenous interior. MRI following stabilization of the patient's general condition revealed necrosis in a substantial portion of the adrenal tumor. ^123^I-MIBG scintigraphy did not reveal uptake at the tumor site, and no excessive catecholamine secretion was detected on multiple urine tests. Accordingly, these findings suggest that the patient experienced excessive catecholamine release and coagulation necrosis of the tumor within five days of the hypertensive crisis.

MIBG is an analog of noradrenaline that can enter chromium-affinity neuroendocrine tumor cells, including pheochromocytomas, neuroblastomas, and paragangliomas, through active uptake via the NAT and passive diffusion (without transporters) [13]. Although ^123^I-MIBG scintigraphy has excellent sensitivity for detecting adrenal pheochromocytomas, it has very low sensitivity for detecting extra-adrenal paragangliomas and metastases (56-75%), which may lead to false-negative findings [14]. Specifically, patients with SDHB mutations often yield false-negative findings as they lack NAT expression [15]. We did not suspect an SDHB mutation as the patient had no history of malignancy, family history of pheochromocytoma, pituitary adenoma, or gastrointestinal stromal tumor. Additionally, calcium antagonists, drugs that can affect MIBG uptake, were discontinued after transfer to our hospital. Therefore, the ^123^I-MIBG false-negative result in our case could be attributed to catecholamine depletion because of extensive necrosis of the tumor cells.

There have been three previously reported cases of ^123^I-MIBG negative results because of pure coagulation necrosis without adrenal bleeding or rupture [16-18] (Table 3).

**Table 3:** Comparison with previously reported cases of MIBG scintigraphy-negative coagulated necrotic pheochromocytoma PASS: pheochromocytoma of the adrenal gland scale

Reference	Sex	Age	Size (CT)	Biochemistry	Symptoms at administration	Cause of necrosis	PASS (20 points maximum)
[16]	M	69	40 mm	24-h urine metanephrine 3.5 mg, 24-h urine normetanephrine 6.0 mg	Headache, palpitation, chest pain	Hypertensive crisis during cardiac catheterization	2
[17]	M	48	60 mm	24-h urine metanephrine 50.53 mg, 24-h urine normetanephrine 13.63 mg	Headache, hypertension	Hypertensive crisis of unknown cause	11
[18]	F	52	25 mm	Plasma adrenaline 0.60 nmol/L, plasma noradrenaline 0.89 nmol/L, 24-h urine adrenaline 0.60 μmol, 24-h urine noradrenaline 0.56 μmol	Transient headache, palpitation, chest pain	No obvious trigger	No listed
This case	F	50	67 mm	24-h urine metanephrine 0.30 mg, 24-h urine normetanephrine 0.94 mg	Nausea, epigastralgia, abdominal pain	Hypertensive crisis aggravated by contrast agent	Unclassifiable

In two cases [16,17], a hypertensive crisis was presumed to underlie the coagulation necrosis. Of these two cases, one [16] exhibited a course similar to the present patient, with alternating hypertension and hypotension causing a crisis during cardiac catheterization performed owing to chest pain. After initiating phentolamine mesylate therapy, the temporary headache, palpitations, chest pain, and blood pressure fluctuations disappeared by day 5 of hospitalization. The other case [18] involved ischemic necrosis in an explanted specimen despite the absence of a dramatic clinical picture that could have triggered necrosis. To the best of our knowledge, this is the first report of pheochromocytoma wherein a hypertensive crisis aggravated by contrast-enhanced CT caused coagulative necrosis of the tumor and negative findings on ^123^I-MIBG scintigraphy.

In the present case, we could not obtain pheochromocytoma of the adrenal gland scale (PASS) and grading of adrenal pheochromocytoma and paraganglioma (GAPP) on histopathological analysis owing to the extensive necrotic tissue on the pathologic picture. It has been reported that approximately 10% of all pheochromocytomas are metastatic. Moreover, predicting the clinical course of individual tumors using scoring systems alone can be challenging [19]. Therefore, both national and international guidelines necessitate permanent postoperative follow-up of all pheochromocytomas.

## Conclusions

We encountered a case of hypertensive crisis aggravated by contrast-enhanced CT, which resulted in coagulation necrosis of the tumor and negative findings on ^123^I-MIBG scintigraphy. ^123^I-MIBG scintigraphy is a useful tool in diagnosing pheochromocytoma; however, a negative result does not rule out pheochromocytoma. Accordingly, clinical judgment must be reached based on the comprehensive assessment of the clinical course and pathological diagnosis, especially for cases involving a hypertensive crisis.
